# One and one makes three—mothers' and fathers' attachment, mentalizing and parenting sensitivity

**DOI:** 10.3389/fpsyg.2025.1582698

**Published:** 2025-07-11

**Authors:** Selina Ismair, Antonia Dinzinger, Gabriela Markova, Jonas Schropp, Karl Heinz Brisch, Wolfgang Sperl, Beate Priewasser

**Affiliations:** ^1^Institute for Early Life Care, Paracelsus Medical University, Salzburg, Austria; ^2^Department of Pediatrics, University Hospital Salzburg, Paracelsus Medical University, Salzburg, Austria; ^3^Inselspital, Bern University Hospital, University of Bern, Switzerland; ^4^Ludwig-Maximilian University of Munich, Munich, Germany

**Keywords:** parenting sensitivity, mentalizing, attachment, family system, actor-partner interdependence model, caregiving, attachment representations, mother and father bidirectional influences

## Abstract

**Objective:**

This study aims to explore how maternal and paternal attachment representations and their reflective functioning (RF), as operationalization of their mentalizing abilities, influence each other's parental sensitivity within a family systems perspective.

**Background:**

Parental sensitivity is crucial for a child's development, and both parental secure attachment and RF are known to enhance sensitive caregiving. However, the impact of one parent's traits and skills on the other's parenting remains unclear.

**Method:**

In a longitudinal, multi-method study of *N* = 40 first-time families, including 40 fathers, mothers and their infants each, we assessed parental attachment during pregnancy using the Adult Attachment Interview (AAI). RF was measured twice, as *general* RF during pregnancy using the RF scale on the AAI, and as *parental* RF when infants were 6 months using the RF scale on the Parent Development Interview. Additionally, parental sensitivity was observed using the Emotional Availability Scales. To investigate associations between parental measures, we employed an actor-partner interdependence model.

**Results:**

We found significant associations between attachment representations and general RF in both mothers and fathers, as well as a mediating effect of fathers' parental RF on the relationship between mothers' general RF and paternal sensitivity. Neither fathers' general RF nor parental RF did influence maternal sensitivity.

**Conclusion:**

The findings suggest interdependencies between mothers' and fathers' reflective functioning and sensitivity, supporting family systems theory. However, given limitations such as a small, homogeneous sample and lack of causal inference, these results should be interpreted cautiously. Yet, the results may have important implications for practice, in that they suggest that both parent's attachment representations and the ability to mentalize in the triadic system should be considered in family interventions.

## 1 Introduction

Sensitive parenting is pivotal for healthy child development (Cooke et al., 2022; Rodrigues et al., 2021). Research grounded in attachment theory has identified key factors and the mechanisms underpinning parental sensitivity. On the one hand, parental ability to mentalize enables parents to relate their children's behavior to their motives, thoughts and feelings and to interpret their behavior accordingly. On the other hand, parental attachment representations seem to be associated with their levels of sensitivity (Zajac et al., 2019). While the sensitivity of parents with insecure-preoccupied or insecure-dismissing representations is lower on average and appears to be dependent on children's arousal levels, parents with secure-autonomous representations interact mostly sensitive, regardless of the child's arousal and attachment signals (van IJzendoorn and Bakermans-Kranenburg, 2019). These associations have been extensively studied in mothers (see Verhage et al., 2016; Zeegers et al., 2017 for meta-analyses), and recent studies also provide first evidence for fathers (e.g., Dinzinger et al., 2023; McFarland-Piazza et al., 2012). However, most studies have examined maternal and paternal sensitivity independently, without considering interdependencies between parents. From a family systems perspective, subsystems within a family (e.g., individual parent-child dyads) are not isolated but mutually influential (e.g., Cox and Paley, 2003). This suggests that one parent's attachment representation and mentalizing abilities may not only shape their own caregiving but are also linked to the other parent's behavior. Therefore, the goal of the present study was to integrate insights form attachment theory and family systems theory to investigate attachment representations and mentalizing of both parents to capture the role of the reciprocal influences and interdependencies on parental caregiving.

When investigating the family as a system, it is essential to recognize the contributions of both, mothers and fathers and to consider the relationship between fathers and children as part of the family system (Bornstein and Sawyer, 2006; Cox and Paley, 2003; Fagan, 2020). This theoretical framework presents a significant challenge in the field, seeing as fathers are underrepresented in research despite their increased involvement in childcare (e.g., Altenburger and Schoppe-Sullivan, 2020; Bakermans-Kranenburg et al., 2019). Therefore, it is important to account for the complex family context by investigating not only mother-child and father-child dyads separately, but also by considering effects of caregivers on each other. These interdependent effects between parental characteristics and skills can be investigated within the family system theory that provides a framework for a holistic and more realistic view of families than traditional investigations of dyadic interactions do (Cox and Paley, 2003). Accordingly, the complexity of family dynamics can be described by three main characteristics: families are units of interdependent individuals, consist of interconnected subsystems (e.g., parent-child, marital, coparenting relationships), and are interactive and living systems with the capacity to adapt and (re-)organize (Holmes and Huston, 2010). Importantly, these influences within a family system are multidirectional (Bornstein and Sawyer, 2006). Thus, adopting a systemic perspective to the study of attachment relationships offers a possible theoretical framework to conceptualize interdependent influences—including direct and indirect effects, such as buffers and vulnerabilities—which may impact child and family outcomes (Cowan, 1997).

Research shows that family dynamics significantly impact individual members and overall family functioning. For instance, parental conflict that leads to stress seems to diminish parents' emotional availability or sensitivity (Bornstein and Sawyer, 2006; Davies et al., 2016). A recent meta-analysis shows that sensitive parenting—the capacity to recognize children's signals, understand their needs and respond to them quickly and appropriately (Ainsworth, 1989)—is correlated between both parents (Deneault et al., 2022), leading the authors to suggest that within the family context, individual parental experiences and behaviors may be transactional (i.e., mutually influential over time). Supporting this idea, a longitudinal study found that sensitive parenting by one parent predicted a positive change in sensitivity by the other parent over time (Scott et al., 2018). Moreover, dyadic and triadic sensitivity are associated (León and Olhaberry, 2020), suggesting that experiences from the triad may spill over to the dyad or vice versa. Overall, sensitive parenting appears to be affected by the family context, including interaction characteristics of other caregivers. Yet, the specific maternal and paternal characteristics associated with each other's sensitivity remain unclear.

Research identified a range of parental attachment-related traits and skills that seem foundational for sensitive parenting, such as secure attachment representations. Numerous studies have shown associations between adult attachment representations (i.e., inner integration of own attachment experiences; Bretherton and Munholland, 2016), sensitive parenting and child attachment patterns (e.g., McFarland-Piazza et al., 2012; van IJzendoorn and Bakermans-Kranenburg, 2019; Verhage et al., 2016). While the different ways in which attachment representations can influence parenting behavior, in accordance with attachment theory (van IJzendoorn and Bakermans-Kranenburg, 2019), are well studied in mothers (e.g., see Sette et al., 2015), only limited and inconclusive evidence exists for fathers (Aytuglu and Brown, 2021; McFarland-Piazza et al., 2012). Similarly, the few studies examining the effect of attachment representations on sensitivity within the family system showed varying results. For example, some studies showed that both parents' secure attachment had the most positive effect on coparenting (McHale, 2007), and predicted more positive caregiving representations in both parents (Psouni, 2019). Other findings suggest that certain combinations are particularly challenging, such as secure mother and insecure father attachment representations, which was found to have the most negative impact on coparenting (Talbot et al., 2009). These findings underscore the necessity for comprehensive investigations that consider both parents' attachment representations, as they jointly contribute to the caregiving environment.

Additionally to attachment representations, mentalizing skills are crucial for sensitive parenting. Mentalizing involves reflecting on one's own and others' mental states to interpret behavior (Fonagy and Allison, 2012) and research showed that mentalizing enables parents to identify their infants' affective cues (Rutherford et al., 2017) while the absence of mentalizing is associated with increased maternal insensitivity (e.g., Ensink et al., 2019; Krink et al., 2018). Indeed, there is meta-analytic evidence for a moderate association between mentalizing and sensitivity in mothers (see Zeegers et al., 2017). Although mentalizing is considered a general characteristic of parent-child interactions (Colonnesi et al., 2019), findings that include male caregivers are scarce and inconsistent. Interestingly, the correlation between mentalizing and sensitivity was shown in interactions between fathers and their children younger than 2 years (e.g., Buttitta et al., 2019; Dinzinger et al., 2023; Planalp et al., 2019), but not with children older than 5 years (Decarli et al., 2023; Gershy and Gray, 2020; Stover and Coates, 2016). Research further suggests that an individual's mentalizing capacity is influenced by the family and can also influence the whole family (Asen and Fonagy, 2012). For instance, families where both parents were insightful, i.e. able to mentalize, were found to have the highest family cooperation and coparenting quality (Marcu et al., 2016). Similarly, maternal mentalizing, as measured by reflective functioning scores, was found to be associated with more positive marital and coparenting interactions, while there was no such relation for fathers (Jessee et al., 2018). Interestingly, low mentalizing only predicted externalizing behavior problems in children at age 4.5 when both parents showed difficulties in mentalizing, suggesting a compensatory effect of one parent's high mentalizing abilities (Colonnesi et al., 2019). Specifically, Bendel-Stenzel et al. (2024) identified a cross-over effect of paternal mentalizing on maternal responsiveness when interacting with their 16-month-old children, but not vice versa. While these findings show the important role of mentalizing in early parent-child interactions, it remains unclear how parents influence each other's mentalizing and caregiving, and how these relations are a function of parents' gender.

Not only do parental attachment representations and their mentalizing influence parental sensitivity, but these two factors are also interdependent. Several studies have shown that secure representations are associated with higher mentalizing while insecure representations might result in lower mentalizing (e.g., Bouchard et al., 2008; Dinzinger et al., 2023; Nazzaro et al., 2017). One potential explanation is that adults' representations of relationships are shaped by how their mental states were addressed in past close relationships (Bretherton and Munholland, 2016), which might impact the value that is placed on one's own and others' thoughts, feelings, and intentions as adults and parents. Indeed, studies have shown that mentalizing mediated the association between attachment and caregiving-related outcomes. For example, using a sample of mothers and fathers, Kungl et al. (2024) found that decreased paternal reflective functioning (prementalizing) partially mediated the association between dismissing attachment representations and reduced supporting presence). Similarly, Nijssens et al. (2018) demonstrated that attachment anxiety was associated with higher parenting stress indices via higher prementalizing in a sample of parent couples. However, the mediating role of mentalizing for the association between attachment and sensitivity in a sample of mothers and fathers has not yet been investigated.

### 1.1 The current study

The goal of the present study was to examine longitudinal and reciprocal effects of maternal and paternal attachment representations, mentalizing, as operationalized through reflective functioning (RF) and parental sensitivity within a single model. By adopting a systemic perspective, we aimed to understand the interdependent and joint effects of parents in their parenting, employing an actor-partner interdependence model (APIM; Cook and Kenny, 2005). We sought to narrow the knowledge gap by focusing on the nuclear family comprising mother, father, and first-born child. To this end, we assessed attachment representation and general RF during pregnancy (T1), and parental RF and sensitivity six months after birth (T2). This approach allowed us to measure the consistency of mentalizing from parents' own childhood experiences to their experiences as parents. Based on the reviewed evidence, we tested the following hypotheses within the APIM framework:

To replicate existing research and ensure a comprehensive analysis, we tested the following Actor associations and effects (1):

H1a: Secure attachment representations in both parents are positively associated with their own sensitivity in parent-child interaction, mediated by their own general and parental RF.

H1b: Higher general RF (T1) in both parents is positively associated with their respective sensitivity in parent-child interactions, with their own parental RF (T2) mediating the association between general RF (T1) and sensitivity.

To examine the interdependencies between parents, we tested the following associations between partners (2) and Partner Indirect (3) Effects:

H2a: RF between parents is associated at both time points.

H2b: One parent's general RF (T1) is associated with the other parent's parental RF (T2).

H2c: One parent's parental RF (T2) is associated with the other parent's sensitivity at T2.

H3: Parental RF (T2) mediates the association between one parent's general RF (T1) and the other parent's sensitivity (T2).

## 2 Methods

### 2.1 Participants

We recruited *N* = 45 couples expecting their first child. Other inclusion criteria were the commitment of both partners to participate, being in the third trimester of pregnancy, carrying a single embryo and speaking German language at time of recruitment. At T2, five families withdrew from the study due to travel distance (*n* = 1), birth complications (*n* = 2) or psychological burden (*n* = 2). We provide box plots of demographic variables in the Supplementary Figure S3 to illustrate potential differences between families that remained in and dropped out of the study. The final sample thus consisted of *n* = 40 fathers (*M*_*age*_ = 32.8 years; *range* = 21–52), 40 mothers (*M*_*age*_ = 31.6 years; *range* = 22–41) and their 40 infants (*M*_*age*_ = 6.5 months; *range* = 5–8; 30% female). Parents were either married (55%) or in a stable relationship (45%) and fathers, in addition to mothers, stayed at home with their child for 4.6 weeks on average. All participants identified themselves as White/Caucasian. A detailed description of demographic information is provided in Table 1.

**Table 1:** Descriptive statistics of demographics for parents and children.

**Demographic variables**	***N***	***%***	***M (range; SD)***
Child's age T2	40		6.45 (5–8; 0.7)
Female children	12	30.0	
**Parental age T1**			
Fathers' age T1	40		32.78 (21–52; 5.8)
Mothers' age T1	40		31.63 (22–41; 3.8)
**Parents' nationality**			
Austria	67	84.0	
Germany	10	12.0	
other	3	4.0	
**Parents' educational attainment**			
Apprenticeship	5	6.5	
A-levels (i.e., high school)	17	21.0	
University degree	56	70.0	
Others	2	2.5	
**Family income per year**			
Low (< 32.000€)	4	5.0	
Lower middle (32.000€−48.000€)	18	22.5	
Middle (48.000€−67.000€)	34	42.5	
Upper middle/high (>67.000€)	24	30.0	
**Parents' attachment representations**			
Both secure	25	62.5	
Both insecure^a^	7	17.5	
Mother secure—father insecure	6	15.0	
mother insecure—father secure	2	5.0	

M, mean; SD, standard deviation. Child age in months, father's age in years. ^a^Insecure attachment comprises both avoidant and preoccupied attachment patterns.

### 2.2 Procedure

We recruited participating families between 2019 and 2021 at birth information events, through flyers at gynecologists and social media ads. Participation was voluntary and monetarily compensated with 30€ per visit. All families participated in a longitudinal investigation including 5 timepoints, but here we describe only procedures of the 2 timepoints relevant to this study. During the third trimester of pregnancy (T1) both parents visited the lab for the Adult Attachment Interview and filled out online questionnaires. Fifteen families were tested before COVID-19 whereas the remaining 25 parents were tested during COVID-19. Families did not differ in our study variables depending on data collection period. Approximately 6 months after birth (T2), videos of parent-infant interactions were recorded in a 360-degree video lab and each parent was administered the Parent Development Interview, in addition to online questionnaires. At this timepoint, only two families were tested before COVID-19. The local ethics committee (Reference number: 415-E/2217/8-2017) approved this study.

### 2.3 Measures

#### 2.3.1 Adult Attachment Interview

During pregnancy, the Adult Attachment Interview (AAI; George et al., 1996) was administered to assess general RF and parental attachment representations. This 1.5–2 h interview includes 20 questions about the respondent's childhood experiences with their parents. Transcribed recordings were coded using the RF scale, which is validated for application to the AAI (Fonagy et al., 1998; Steele and Steele, 2008) and measures the ability to reflect on one's own and others' thoughts, feelings and motives and to use these internal mental states to explain behavior. The scores are ranging from −1, which represents negative RF (e.g., anti-reflective, bizarre, or inappropriate responses) to 9, which represents optimal or excellent RF. Additionally, we used the AAI manual (Main and Goldwyn, 1994) to categorize attachment representations as secure (F), dismissive (Ds), enmeshed (E), or unresolved (U). Due to the small number of cases, we used the dichotomous categorization of secure (F) and insecure (Ds, E, U) attachment representations for analyses. A reliable master coder performed all RF and attachment ratings, with 10% double ratings for attachment representations performed by a second reliable coder, who agreed in all cases (κ = 1) and 20% double codings for RF performed by a third reliable coder, who also showed very good agreement (ICC = 0.80). All three raters were blinded to participant characteristics.

#### 2.3.2 Parent Development Interview

We assessed parental RF as operationalization for parental mentalizing using the Parent Development Interview (PDI-R; Slade et al., 2022) at T2. The short version of the semi-structured interview consists of 30 questions that address the parenting self and the parents' current relationship with their children. The questions are specifically worded to give the interviewees the opportunity to respond in a reflective manner (e.g., describe a situation where parents were really angry or upset). The interviews lasted approximately 1 hour, were audiotaped and the verbatim was transcribed. To analyze parental RF, transcripts were coded using the Addendum to the Reflective Functioning Scoring Manual, which is an adaptation of the original RF scale for application to the PDI, to specifically capture parents' reflective functioning in relation to their own child and their experiences as parents (Slade et al., 2022). The range of the RF scores is the same as described above for the AAI (Fonagy et al., 1998). The scores were coded by two independent reliable coders, each of whom coded 60% of the interviews, resulting in a 20% overlap that was used for double-ratings. Interrater-reliability was satisfactory, with ICC = 0.86 overall (ICC for fathers = 0.62; ICC for mothers = 0.92).

#### 2.3.3 Emotional Availability Scales

Parental sensitivity was assessed at T2 by videotaping 20-minute interactions between each parent and their child, typically conducted in a face-to-face setting, with the infants seated in a bouncer. Occasionally, due to the infants' dissatisfaction or falling asleep, the situation had to be slightly modified (e.g., interaction on the floor, or shorter interaction time). Videos were scored using the Emotional Availability Scales (EAS, Biringen, 2008), which capture six dimensions: sensitivity, structuring, non-intrusiveness, and non-hostility, as well as child responsiveness and involvement. Each dimension receives a *direct score* (1–7) and includes seven subscales, scored on a 1–7 or 1–3 scale, which are summed for a *total* dimension score. For this study, we used the direct sensitivity score, which captures warmth, congruence and responsiveness. Three independent raters, reliably trained by Zeynep Biringen on the *direct* scores, coded the videos, with 20% double-coded for interrater reliability. ICCs of 0.83 for all videos (ICC = 0.85 for mothers and ICC = 0.80 for fathers) indicate a satisfactory interrater agreement.

### 2.4 Data analysis

Calculations were performed using R version 4.3.1 Patched—”Beagle Scouts,” using lavaan 06-16 for SEM and boot 1.3.28.1 for bootstrapping inference (Rosseel, 2012), and were done only for families with complete data (i.e., T1 and T2).

To answer our hypotheses, we used Pearson's correlations (*r*) and a structural equation model (SEM). Correlations, including 95% confidence intervals and *p*-values are based on the bias-controlled and accelerated bootstrapping to account for non-normality or outliers. SEMs are based on the APIM (Cook and Kenny, 2005), which was extended by including RF at multiple time points. Residual covariances were used to model additional unexplained variance between partners at the same time point, while effects from T1 to T2 were always directed. We did not include the measurement model and treated scales as observed due to power concerns. This approach offers flexibility in defining individual effects as well as interactions between the two parents. The final model constrained the standardized actor and partner effects between RF at T1 and T2 to be equal across parents, as well as the path from attachment representations to RF at T1 and the covariance between maternal and paternal attachment representations, based on fit indices (CFI, TLI, RMSEA) and consistency with expected psychological pathways. That is, if the same effect for mothers and fathers significantly worsened the model—estimated using the fit indices—then the paths were allowed to vary, otherwise not. We present both the completely unrestrained as well as the completely restrained models in the Supplementary material. Maximum likelihood estimation was used with coefficients and confidence intervals based on the bias-corrected bootstrap percentile methods. Decisions to reject the null hypothesis were based on bias corrected 95% confidence intervals. We report correlations (*r*) of the unadjusted analysis and fully standardized path coefficients (β) as well as results on the original scale (*b*) for the SEM. Mediation effects were calculated by taking the product of the involved paths.

We used a random seed of 101 for reproducibility and 10,000 bootstrapping replicates for all analyses. The overall model fit was evaluated based on the cutoffs defined in Hu and Bentler (1999), using the Chi^2^-test, CFI, TLI (both ideally ≥ 0.95) and RMSEA (ideally ≤ 0.06).

## 3 Results

### 3.1 Preliminary analyses

Differences between mothers and fathers on the variables of interest are presented in Table 2, showing that parental reflective functioning and sensitivity were significantly higher for mothers. We found no associations between demographic characteristics (age, education, income, child gender) and the study variables, with the exception of maternal education, which was significantly correlated with multiple study variables. Additionally, maternal sensitivity was significantly correlated with maternal age (*r* = 0.39; *p* < 0.001), and paternal age (*r* = 0.31; *p* < 0.001). We found significant correlations in the hypothesized directions between all variables of interest, except for a lack of associations between maternal sensitivity and any other variables (*p* > 0.05; see Table 3) than for fathers.

**Table 2:** Differences between mothers and fathers.

**Variables**	**Mothers (*n =* 40)**	**Fathers (*n =* 40)**	***T*** **(d*****f*****)**	***p***
	***M*** **(SD)**	***M*** **(SD)**		
RF_AAI	5.66 (1.30)	5.10 (1.50)	1.79 (78)	0.077
RF_PDI	5.35 (1.17)	4.43 (1.7)	2.81 (68.6)	0.006
Sensitivity	5.66 (1.12)	4.95 (1.17)	2.78 (78)	0.007
Secure attachment	*n* (%) 31 (77.5)	*n* (%) 27 (67.5)	χ^2^ 1.00	

RF_AAI, general reflective functioning assessed with the AAI at T1. RF_PDI, parental reflective functioning assessed with the PDI at T2. Sensitivity assessed with EAS at T2. Secure attachment assessed with the AAI at T1.

**Table 3:** Correlations of demographics, attachment representation, general and parental reflective functioning and sensitivity for mothers and fathers.

**Variables**	**Descriptive statistics**	**[2]**	**[3]**	**[4]**	**[5]**	**[6]**	**[7]**	**[8]**	**[9]**	**[10]**	**[11]**	**[12]**	**[13]**
[1] Age (F)	32.8 (5.8)	0.61^**^	0.27	0.4^**^	0.29^*^	0.14	0.31^**^	0.06	−0.04	−0.02	−0.09	0.08	0.24
[2] Age (M)	31.6 (3.8)		0.11	0.13	0.21	0.26	0.39^**^	−0.01	−0.18	0.06	−0.16	0.13	0.18
[3] Annual net income				0.19	0.19	0.2	0.11	0.21	0.07	0.09	0.03	0.06	0.11
[4] Education (F)					0.46^*^	0.12	0.06	0.26	0.12	0.19	−0.02	0.23	0.26
[5] Education (M)						0.25	0.24	0.48^**^	0.44^*^	0.42^**^	0.3^*^	0.44^**^	0.44^**^
[6] Sensitivity (F)	4.95 (1.17)						0.27	0.34^*^	0.29^*^	0.63^**^	0.1	0.36^**^	0.34^*^
[7] Sensitivity (M)	5.66 (1.12)							0.03	0.11	0.22	0.2	0.07	0.21
[8] Secure AR (F)	27 (68%)								0.52^**^	0.55^**^	0.26	0.75^**^	0.61^**^
[9] Secure AR (M)	31 (78%)									0.35^*^	0.22	0.28	0.67^**^
[10] RF (F, PDI)	4.43 (1.72)										0.51^**^	0.59^**^	0.52^**^
[11] RF (M, PDI)	5.35 (1.17)											0.45^**^	0.43^**^
[12] RF (F, AAI)	5.10 (1.50)												0.58^**^
[13] RF (M, AAI)	5.66 (1.30)												

*N* = 40. Variables for mothers are indicated with “M” and for fathers with “F.” AR, Attachment representations (secure vs. insecure), assessed with the AAI at T1. RF_AAI, Reflective functioning assessed with the AAI at T1. RF_PDI, Reflective functioning assessed with the PDI at T2. Sensitivity was measured using the EAS at T2. The correlation matrix includes variables with different measurement scales: continuous (Age, RF_AAI, RF_PDI, Sensitivity), ordinal (Family Income, Education), and nominal (AR). Descriptive statistics represent M (SD) for continuous/ordinal variables and *N* (%) for dichotomous variables Pearson's correlation was used for continuous-continuous variables, Kendall's tau for correlations involving ordinal variables, and point-biserial correlation for dichotomous-continuous variables. *P*-values calculated using 2000 bootstrap replicates.

^*^p < 0.05; ^**^p < 0.001.

### 3.2 Hypotheses

**Figure 1:**
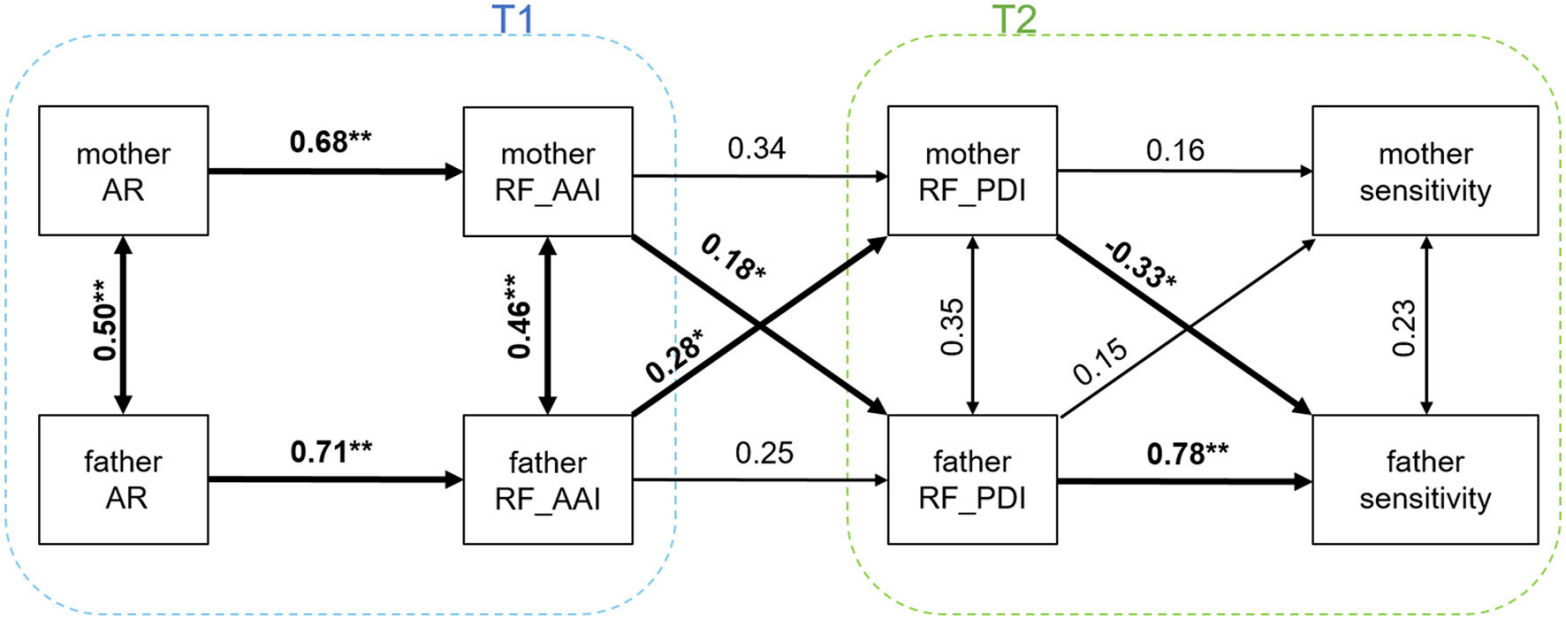
Actor-partner interdependence model with standardized path coefficients. Squares represent observed variables (i.e., scale scores without estimating factor loadings); AR, adult attachment representations; RF_AAI, general reflective functioning measured with the AAI; RF_PDI, parental reflective functioning measured with the PDI; *p < 0.05; **p < 0.001; Even though the pathways are partially constrained, the coefficients for the maternal and paternal pathways may still be slightly different due to different means and standard deviations for mothers and fathers. Model fit: χ^2^(17) = 15.34, *p* = 0.571; CFI = 1.00; TLI = 1.02; RMSEA = 0.00, 90% CI [0.00, 0.13].

The SEM fit well to the data (*CFI* = 1, *TLI* = 1.021). The RMSEA approached 0, but its 95% CI was wide ranging between 0 and 0.13. The model did not significantly deviate from the observed data with χ^2^(17) = 15.34 (*p* = 0.571). In terms of comparative fit, it outperformed both the fully unconstrained model (AIC = 821, BIC = 875) and the fully constrained model (AIC = 829, BIC = 859), with lower AIC = 813 and equivalent BIC = 859, suggesting similar parsimony but better model-data fit. The SEM results are visualized in Figure 1. To support findings from the SEM results, Table 3 shows correlations between all relevant demographic and study variables of both mothers and fathers. Below, we primarily report the results from the APIM-based SEM, which accounts for shared variance between partners and offers estimates of unique actor and partner effects. To provide additional descriptive context, we also report relevant bivariate correlations; however, these are unadjusted and therefore should not be interpreted as equivalent to the SEM results.

*Actor associations and effects* describe pathways between individual variables within the same person (e.g., mother's attachment representation and her reflective functioning). In line with our hypotheses, the APIM model revealed a significant indirect (mediated) actor effect for fathers: the association between their attachment representations and sensitivity was sequentially mediated by their own general RF (T1) and parental RF (T2), β = 0.14, *b [95% CI]* = 0.34 [0.01; 0.79]. Consistent with this, the indirect path from fathers' general RF at T1 via fathers' parental RF at T2 to fathers' sensitivity was also significant: β = 0.20, *b [95% CI]* = 0.16 [0; 0.35]. No such effects were found for mothers (β = 0.03 and 0.04, respectively; both non-significant). Complementing these findings, *z*ero-order (bivariate) correlations showed that fathers' attachment representations were significantly correlated with their sensitivity, *r* = 0.34, *p* = 0.015, while the corresponding correlation was not significant for mothers. Similarly, for fathers, both general RF (T1) and parental RF (T2) were significantly correlated with sensitivity (*r* = 0.36 and *r* = 0.63, respectively), whereas these associations were not significant for mothers.

*Partner associations and effects* describe pathways where an individual variable of one parent predicts an individual variable of the other parent (e.g., father's RF and mother's sensitivity) and associations between partner's characteristics at the same time point RF between the two parents was significantly associated at both T1 (*r* = 0.582, *95% CI* = [0.301; 0.754], *p* < 0.001), and T2 (*r* = 0.511, *95% CI* = [0.243; 0.701], *p* = 0.001). There was also significant residual covariance in the SEM for T1 (β = 0.46, *b [95% CI]* = 0.43 [0.18; 0.76], *p* = 0.003) but not for T2 (β = 0.35, *b [95% CI]* = 0.46 [0.01; 1.04], *p* = 0.075). Next, we tested whether one parent's general RF (T1) was associated with the other parent's parental RF (T2). The SEM revealed significant partner effects in both directions: from fathers' general RF to mothers' parental RF (β = 0.28, *b [95% CI]* = 0.23 [0.02; 0.44], *p* = 0.03), and from mothers' general RF to fathers' parental RF (β = 0.18, *b [95% CI]* = 0.23 [0.02; 0.44], *p* = 0.03). Finally, we examined whether one parent's parental RF (T2) was associated with their partner's sensitivity (H2c). In contrast to our expectations, fathers' parental RF was not significantly associated with mothers' sensitivity (β = 0.15, *p* = 0.356), and mothers' parental RF was negatively related to fathers' sensitivity (β = −0.33, *b [95% CI]* = −0.33 [−0.68; −0.05], *p* = 0.04). Complementing these findings, bivariate correlations between relevant partner variables showed that general RF at T1 in one parent was significantly associated with parental RF at T2 in the other parent: fathers' general RF was correlated with mothers' parental RF, *r* = 0.449, *95% CI* = [0.134; 0.655], *p* = 0.002, and vice versa for mothers' general RF and fathers' parental RF, *r* = 0.522, *95% CI* = [0.252; 0.700], *p* < 0.001. In contrast, no significant correlations were found between one parent's parental RF and the other parent's sensitivity: father's parental RF with mother's sensitivity, *r* = 0.215, *95% CI* = [−0.146; 0.501], *p* = 0.216; and mother's parental RF with father's sensitivity, *r* = 0.098, *95% CI* = [−0.207; 0.363], *p* = 0.496.

*Partner indirect effects*. These effects describe pathways where an individual variable of one parent affects an individual variable of the other parent via an intermediate variable, indicating that the influence does not occur directly but rather through a mediating factor. We expected that the path between an actor's general RF (T1) to their respective partner's sensitivity (T2) would be mediated by the partner's parental RF (T2). We found that general RF (T1) of mothers was positively related to fathers' parental RF (T2), which in turn was positively associated with fathers' sensitivity at T2 (β = 0.14, *b [95% CI]* = 0.12 [0.02; 0.26]). The indirect path from fathers' general RF (T1) via mothers' parental RF (T2) to mothers' sensitivity was not confirmed (β = 0.03, *b [95% CI]* = 0.04 [−0.04; 0.13]).

All additional path coefficients of the SEM, which were not part of the main hypotheses, are displayed in Figure 1.

## 4 Discussion

The present study examined the interdependent effects of attachment related traits and skills of both parents in a single model. We found that father's attachment representation was related to his sensitivity in interaction with the infant, and mediated by general and parental RF pre- and postnatally (i.e., actor effect for fathers, which could not be confirmed for mothers). Similarly, we found a correlation between both general and parental RF and sensitivity, as well as an indirect effect of general RF prenatally via parental RF postnatally on sensitivity for fathers only. Maternal and paternal RF were correlated with each other at each of the time points, and fathers' general RF was associated with mothers' parental RF and vice versa (partner effects). While we found no significant unadjusted correlations between one parent's general nor parental RF and the respective other parent's sensitivity, there was a significant negative path from mother's parental RF to father's sensitivity, contrary to our expectations. Finally, analyses showed an indirect partner effect where maternal general RF may indirectly enhance fathers' sensitivity by improving their parental RF. We did not find this effect for the path from maternal RF to paternal sensitivity.

As expected, data analyses showed associations between paternal attachment, general and parental mentalizing, and sensitivity. This is consistent with recent studies on fathers (e.g., Buttitta et al., 2019; León and Olhaberry, 2020; McFarland-Piazza et al., 2012) and provides much needed evidence in the still neglected field of paternal attachment research. Surprisingly, our findings did not show any associations between maternal attachment, mentalizing and maternal sensitivity, which contrasts with a recent study by Mattheß and colleagues who found associations between maternal attachment experiences, reflective functioning and sensitivity, but no moderating effects of reflective functioning for mothers (Mattheß et al., 2024). Moreover, our results differ from recent meta-analyses indicating associations between these factors (Verhage et al., 2016; Zeegers et al., 2017). The results of the meta-analyses are largely based on investigations of parental mentalizing operationalized through mind-mindedness (see Zeegers et al., 2017), while in our study we utilized reflective functioning as a measure of mentalizing, which may capture distinct aspects of parental mentalizing (Dollberg, 2021). Mind-mindedness, often assessed through direct observations, may be more sensitive to momentary and implicit aspects of mentalizing, whereas reflective functioning, measured through retrospective narratives, assesses the inner representation of the child and oneself, requiring a more controlled and explicit meta-cognitive effort (Dollberg, 2021; Zeegers et al., 2017). In line with the proposed different dimensions and facets of mentalizing (e.g., Luyten et al., 2020), one could argue that mind-mindedness captures more automatic processes and reflective functioning reveals more controlled facets. Interestingly, several studies did not find associations between mind-mindedness and sensitivity/responsiveness in samples with fathers (Ierardi et al., 2023; Miller et al., 2019; Planalp et al., 2019), but associations between reflective functioning and father-child interaction quality (see Charpentier Mora et al., 2023). It could be that implicit, automatic mentalizing plays a more important role for mother-child interactions (Dollberg, 2021; Shai and Belsky, 2011) potentially facilitated by neurobiological and hormonal adaptations linked to pregnancy and breastfeeding (e.g., Feldman, 2015; Kim et al., 2010), which may enhance automatic, implicit forms of mentalizing relevant for caregiving. In contrast, explicit, controlled mentalizing could be a better explanatory factor for fathers' sensitivity. Future studies need to investigate the neurocognitive and psychological mechanisms underlying the observed differences in maternal and paternal dimensions of mentalizing and sensitivity.

Addressing a gap in existing research (Camoirano, 2017), our results from the bivariate correlations showed associations between general and parental reflective functioning pre- and postnatal for both mothers and fathers. However, these associations did not remain significant in the SEM, suggesting that once other pathways and partner effects were accounted for, the direct link between general RF on parental RF may be less robust. These discrepant findings could imply that while a parent's ability to reflect on their own and their caregiver's inner world when discussing their own childhood may be related to their capacity to adopt a mentalizing attitude toward their children, this relationship is likely influenced by additional contextual or interpersonal factors and is not solely deterministic. Our findings further revealed that mothers had higher levels of reflective functioning than fathers on the PDI, measuring parental aspect of reflective functioning, but not in the AAI, capturing a more general aspect of this construct. However, compared to other studies, we found that the mean parental reflective functioning in fathers in our study (*M* = 4.4) was still higher than in similar studies (e.g., *M* = 3.1–3.8, see Buttitta et al., 2019; Decarli et al., 2023; León and Olhaberry, 2020; Stover and Coates, 2016), suggesting that this difference is likely due to the exceptionally high maternal reflective functioning in our study rather than low paternal abilities.

Confirming previous assumptions of parental interdependence (e.g., Colonnesi et al., 2019; Marcu et al., 2016), we found that maternal and paternal reflective functioning were related within and across time. Specifically, bivariate correlations showed associations between parents' mentalizing at the same time, as well as a temporal link between prenatal and postnatal mentalization. Importantly, the SEM extended these findings by accounting for partner effects and temporal dependencies, revealing significant associations between maternal and paternal RF during pregnancy, along with cross-lagged partner effects from prenatal maternal RF to postnatal paternal RF and vice versa. However, the residual covariance between maternal and paternal RF at the postnatal stage was not significant, suggesting that their concurrent association is fully accounted for by prior partner and actor effects. These patterns align with the inconsistencies reported in previous research (e.g., Gershy and Gray, 2020; Jessee et al., 2018; León and Olhaberry, 2020). This may indicate that partner influences on mentalizing may be particularly salient during the transition to parenthood. As discussed earlier, studies employed different measures capturing different dimensions of mentalizing (Luyten et al., 2020), suggesting that some facets of mentalizing may be more susceptible to influence from a partner than others. Furthermore, the different phases of parenthood when mentalization is assessed (i.e., pregnancy, Jessee et al., 2018; infancy, León and Olhaberry, 2020; early to middle childhood, Gershy and Gray, 2020) may contribute to inconsistent findings, as the capacity to mentalize develops throughout life and in interaction with others (e.g., one's own children; Luyten et al., 2020). Finally, to the best of our knowledge, previous studies have only considered cross-sectional correlations, whereas our study examined longitudinal associations between mothers' and fathers' mentalizing over the transition to parenthood. Further investigations are needed to generalize these findings across different stages of parenting and measures of mentalizing.

Importantly, we found an association between maternal prenatal general reflective functioning and paternal sensitivity, mediated by fathers' postnatal parental reflective functioning, supporting the assumptions that mothers play a significant role in shaping fathers' parenting behavior, and that fathers are more impacted by their coparent than vice versa (e.g., Le et al., 2017). Traditional socialization patterns often hinder men's full engagement in caregiving roles compared to the stronger socialization women receive in this area (McGill, 2014). Thus, women likely take on the role of the primary caregiver while males “follow the lead”. The observed differences could then not be attributed to gender but to the specific parental role. Further research, particularly in families where fathers are primary caregivers (e.g., gay families, stay-at-home dads) is needed for a more nuanced understanding.

While our findings reveal a connection between maternal and paternal reflective functioning, they also challenge existing assumptions about the link between maternal and paternal sensitivity. The lack of a significant association in the present study contradicts recent studies and meta-analyses showing that maternal and paternal sensitivity are related (e.g., Deneault et al., 2022; Scott et al., 2018). However, the association found in the meta-analysis can be considered moderate (*r* = 0.23; Deneault et al., 2022) and would require a sample of at least 194 parents to find. Therefore, the lack of association in our study should be interpreted with caution as this may be due to the limited sample size as well as the influence of various individual characteristics, such as parental personality, child characteristics, and other family macrosystem factors (e.g., Cabrera et al., 2014) on parental sensitivity.

More unexpectedly, the SEM revealed a negative association between maternal RF at T2 and paternal sensitivity, despite no significant bivariate correlation between these variables. This pattern may reflect a suppression effect arising from statistical control: once paternal RF is accounted for, higher maternal RF is associated with lower paternal sensitivity. One possible interpretation may be that this reflects compensatory dynamics in caregiving—for example, in families where mothers exhibit higher reflective functioning, fathers may step back from sensitivity-related roles due to reduced need or opportunity, or vice versa. Alternatively, this effect may reflect unmeasured third variables, such as co-parenting quality, power dynamics, or parental stress. Further research is needed to disentangle the complex interplay between parental reflective functioning and sensitivity and to explore potential mediating or moderating family system factors.

The present study is subject to certain limitations. The selective inclusion criteria such as cohabiting first-time parenthood and low-risk pregnancy, as well as a homogeneous sample, with more than 70% of families having medium to high income and 70% of the mothers holding a university-level degree, could limit the generalizability of our findings to the broad spectrum of families. Furthermore, when interpreting the results, it is important to consider that although maternal education was found to correlate with all outcome factors, it was not included in the statistical model to avoid overfitting and excessive model complexity given the limited sample size. This decision, alongside the socioeconomic bias in our sample, further restricts the transferability of our findings to more diverse populations. Moreover, even though the reflective functioning scale is considered gold standard (Taubner et al., 2013), this trait-like scale may provide less information about subtle mentalizing difficulties across multiple facets (see Fonagy and Luyten, 2009; Taubner, 2020). New measures focusing on different facets of mentalizing would be needed for a more nuanced assessment (Taubner, 2020). Similarly, although the AAI is considered the gold standard for assessing adult attachment, the small sample sizes in each of the four categories in our study necessitated a dichotomous classification (secure vs. insecure). While this approach is common in attachment research (e.g., in Aytuglu and Brown, 2021; McFarland-Piazza et al., 2012) and considered an economical solution (Bakermans-Kranenburg and van IJzendoorn, 2009), it loses important information and compromises interpretability. Given the distinct phenotypic and qualitative differences among dismissing, preoccupied, and unresolved attachment representations, aggregating parents into a single “insecure” category may result in a highly heterogeneous group and therefore should be accounted for in future research. Furthermore, both parent attachment representations and general reflective functioning were coded from the same narrative material of the AAI. While this is a practice employed in several prior studies (e.g., Nazzaro et al., 2017; Rosso, 2022), the use of a shared source may have inflated associations between constructs due to common method variance, again highlighting the need for multi-method approaches in future research. A notable limitation concerns the interrater reliability of RF ratings on the PDI, which, although satisfactory overall (ICC = 0.86), was substantially lower for fathers (ICC = 0.62) than for mothers (ICC = 0.92). According to commonly accepted thresholds, this ICC would be considered “good” but not “excellent,” and may have introduced measurement error into paternal RF scores (Cicchetti, 1994). This could have attenuated associations involving paternal mentalization. The discrepancy in ICCs for mothers' and fathers' interviews may reflect greater variability in fathers' narratives or coding challenges, highlighting the need for more targeted training for coders who work with fathers or adapted coding frameworks in future studies. Additionally, although our study includes longitudinal data, it does not permit conclusions about causality. The simultaneous assessment of postnatal mentalizing and sensitivity introduces ambiguity about their temporal relation, and our mediation model might be affected by reverse causation. In terms of statistical power, a larger sample size would have been needed to detect smaller combined effects. The present sample was small relative to the complexity of our theoretical model, and the wide confidence interval of the RMSEA (0; 95% CI = [0; 0.13]) indicates that the model might not be stable and potentially may not generalize well to unseen data. Therefore, the results of the APIM should be interpreted with caution. Given that the present study focused on the transition to parenthood (i.e., a vulnerable period in a family's life; Bornstein and Sawyer, 2006) and our data suggest that high prenatal maternal mentalizing may be a key factor in coping with this transition, future studies need to incorporate child outcomes to better represent the entire family and directly assess the impact of the family.

Results of the present study have significant practical implications for family screenings and interventions. Importantly, our findings suggest that factors such as mentalizing capacity and attachment representations assessed during pregnancy may predict later parental mentalizing and sensitivity, particularly for fathers. Identifying impairments in these abilities early on offers an opportunity for targeted intervention. While the SEM did not indicate longitudinal stability of reflective functioning within individual parents (i.e., from general to parental RF), it did reveal partner effects already during pregnancy and longitudinally between both parents' general and parental RF. In combination with studies demonstrating the benefits of early onset prevention, ideally beginning during pregnancy (e.g., Heckman et al., 2013; Walter et al., 2019), findings of the present study support the relevance of early family-centered interventions that address both partners, even before the child is born. To achieve more lasting effects on parental functioning and the family system, such interventions should not be limited to the prenatal period but should extend into the postpartum phase, when parenting demands and individual differences in caregiving become more salient (e.g., Slade et al., 2019). Based on the results of the SEM, it is possible that enhancing one parent's mentalization skills will positively impact the other parent's attachment related abilities. However, the majority of prevention and intervention initiatives only target mothers, despite evidence indicating that interventions are similar or even more effective when both partners are involved (e.g., C. P. Cowan and Cowan, 2019; McKee et al., 2021; Panter-Brick et al., 2014). The identification of maternal mentalizing playing a key role in the family system highlights the benefit of focusing on attachment related traits and skills of both parents. However, with only one mentalizing program to our knowledge including both parents and already starting during pregnancy (SAFE^®^ Brisch, 2024; Walter et al., 2024), preventive interventions targeting attachment related traits and skills while involving both parents are still rare.

Our study makes two key contributions to family research. First, by including both mothers and fathers, we address the ongoing underrepresentation of fathers in family and attachment studies, highlighting their crucial role as attachment figures (e.g., Bretherton, 2011) and the need to regularly include them to fully understand their impact on child development. Second, by examining the interdependent effects within the nuclear family system we emphasize the importance of considering the ecological context when studying attachment (Volling and Palkovitz, 2021). Despite its limitations, this study represents a significant step toward understanding the complex interplay of attachment-related traits and skills in both parents during the critical transition to parenthood. By addressing cross-over effects and longitudinal associations, our work enhances ecological validity and informs the development of more comprehensive, culturally sensitive interventions to support families.

## Data Availability

The datasets presented in this study can be found in online repositories. The names of the repository/repositories and accession number(s) can be found below: https://github.com/jonas-schropp/one-and-one-makes-three.
