# Supplementary material for: One and one makes three—mothers' and fathers' attachment, mentalizing and parenting sensitivity
Source: Front Psychol. 2025 Jul 11;16:1582698. doi: 10.3389/fpsyg.2025.1582698 (PMC12289603; doi:10.3389/fpsyg.2025.1582698)

One and One Makes Three – The Mutual Influence of Mothers' and Fathers' Attachment, Mentalizing, and Parenting Sensitivity

**Supplementary Material**

**Table S1**. Results of the partially restrained structural equation model for attachment representation (AR), reflective functioning (RF), and sensitivity in the parent-child interaction (Model 1).

| path | parent | ß | *b* [95% CI] | *se* | *p* |
| --- | --- | --- | --- | --- | --- |
| RF (T1), int. | F | 2.62** | 3.68 [3.26; 4.13] | 0.23 | <.001 |
| RF (T1)<--Secure AR (T1) | F | 0.71** | 2.11 [1.57; 2.62] | 0.27 | <.001 |
| RF (T1), int. | M | 3.1** | 4.03 [3.54; 4.51] | 0.25 | <.001 |
| RF (T1)<--Secure AR (T1) | M | 0.68** | 2.11 [1.57; 2.62] | 0.27 | <.001 |
| RF (T2), int. | F | 0.54 | 0.9 [-0.44; 2.22] | 0.68 | .186 |
| RF (T2)<--RF (T1) | F | 0.25 | 0.3 [-0.01; 0.62] | 0.16 | .067 |
| RF (T2)<--RF (T1) | F<-M | 0.18* | 0.23 [0.02; 0.44] | 0.11 | .03 |
| RF (T2)<--Secure AR (T1) | F | 0.3* | 1.04 [0.03; 2.02] | 0.51 | .04 |
| RF (T2), int. | M | 2.36** | 2.69 [1.28; 4.16] | 0.73 | <.001 |
| RF (T2)<--RF (T1) | M | 0.34 | 0.3 [-0.01; 0.62] | 0.16 | .067 |
| RF (T2)<--RF (T1) | M<-F | 0.28* | 0.23 [0.02; 0.44] | 0.11 | .03 |
| RF (T2)<--Secure AR (T1) | M | -0.1 | -0.26 [-1.21; 0.69] | 0.48 | .591 |
| Sensitivity (T2), intercept | F | 3.8** | 4.35 [2.99; 5.65] | 0.67 | <.001 |
| Sensitivity (T2)<--RF (T2) | F | 0.78** | 0.54 [0.35; 0.71] | 0.09 | <.001 |
| Sensitivity (T2)<--RF (T2) | F<-M | -0.33* | -0.33 [-0.68; -0.05] | 0.16 | .04 |
| Sensitivity (T2), int. | M | 3.88** | 4.35 [2.99; 5.65] | 0.67 | <.001 |
| Sensitivity (T2)<--RF (T2) | M | 0.16 | 0.16 [-0.22; 0.44] | 0.17 | .339 |
| Sensitivity (T2)<--RF (T2) | M<-F | 0.15 | 0.1 [-0.13; 0.3] | 0.11 | .356 |
| Secure AR (T1), int. | F | 1.56** | 0.74 [0.59; 0.86] | 0.07 | <.001 |
| Secure AR (T1), int. | M | 1.76** | 0.74 [0.59; 0.86] | 0.07 | <.001 |
| RF (T2)<-->RF (T2) | F<->F | 0.63** | 1.74 [1.03; 2.99] | 0.46 | <.001 |
| RF (T2)<-->RF (T2) | M<->M | 0.77** | 1 [0.71; 1.56] | 0.20 | <.001 |
| RF (T1)<-->RF (T1) | F<->F | 0.5** | 0.97 [0.63; 1.42] | 0.20 | <.001 |
| RF (T1)<-->RF (T1) | M<->M | 0.54** | 0.91 [0.56; 1.39] | 0.21 | <.001 |
| Sensitivity (T2)<-->Sensitivity (T2) | F<->F | 0.54** | 0.71 [0.51; 1.04] | 0.13 | <.001 |
| Sensitivity (T2)<-->Sensitivity (T2) | M<->M | 0.93** | 1.16 [0.85; 1.61] | 0.19 | <.001 |
| Secure AR (T1)<-->Secure AR (T1) | F<->F | 1** | 0.22 [0.15; 0.27] | 0.03 | <.001 |
| Secure AR (T1)<-->Secure AR (T1) | M<->M | 1** | 0.18 [0.09; 0.23] | 0.04 | <.001 |
| RF (T2)<-->RF (T2) | F<->M | 0.35 | 0.46 [0.01; 1.04] | 0.26 | .075 |
| RF (T1)<-->RF (T1) | F<->M | 0.46** | 0.43 [0.18; 0.76] | 0.15 | .003 |
| Secure AR (T1)<-->Secure AR (T1) | F<->M | 0.5** | 0.1 [0.04; 0.17] | 0.03 | .004 |
| Sensitivity (T2)<-->Sensitivity (T2) | F<->M | 0.23 | 0.2 [-0.03; 0.52] | 0.14 | .138 |
| h1a_father | h<-s | 0.14 | 0.34 [0.01; 0.79] | 0.19 | .084 |
| h1a_mother | h<-s | 0.03 | 0.1 [-0.08; 0.48] | 0.13 | .451 |
| h1b_father | h<-s | 0.2 | 0.16 [0; 0.35] | 0.09 | .066 |
| h1b_mother | h<-s | 0.04 | 0.05 [-0.04; 0.21] | 0.06 | .435 |
| h3_father | h<-r | 0.14* | 0.12 [0.02; 0.26] | 0.06 | .048 |
| h3_mother | h<-r | 0.03 | 0.04 [-0.04; 0.13] | 0.04 | .395 |
| ꭕ^2^(17) =15.342 (p=0.571); *CFI*=1; *TLI*=1.021; *RMSEA*=0 95%; CI [0; 0.13]; *N*=40 | | | | | |
| Note. F = father, M = mother. ß is the path coefficient with all variables standardized; *b* [95% CI] is the unstandardized path coefficient with a 95% bias-corrected and accelerated confidence interval; *se* is the bootstrapped standard error the estimate; int. is the intercept of the path;**p* < .05; ** *p* < .01; all calculations are based on 10.000 bootstrap replicates. *h1a_father* = Indirect effect of father attachment via father RF_AAI via father RF_PDI on father sensitivity; *h1a_mother* = Indirect effect of mother attachment via mother RF_AAI via mother RF_PDI on mother sensitivity; *h1b_father* = Indirect effect of father RF_AAI via father RF_PDI on father sensitivity; *h1b_mother* = Indirect effect of mother RF_AAI via mother RF_PDI on mother sensitivity; *h3_father* = Indirect effect of mother RF_AAI via father RF_PDI on father sensitivity; *h3_mother* = Indirect effect of father RF_AAI via mother RF_PDI on mother sensitivity. | | | | | |

**Table S2.** Differences between the partially constrained (Model 1) and the unconstrained model (Model **2**).

| outcome | predictor | Effect constrained |
| --- | --- | --- |
| secure attachment | - | Mean level (intercept) constrained to be the same for fathers and mothers |
| RF (T1) | attachment | Actor effects constrained to be the same for fathers and mothers |
| RF (T2) | RF (T1) | Actor effects constrained to be the same for fathers and mothers |
| RF (T2) | RF (T1) | Partner effects constrained to be the same for both father to mother and mother to father |
| RF (T2) | attachment | Actor effects constrained to be the same for fathers and mothers |

**Table S3**. Results of the fully unconstrained structural equation model for attachment representation (AR), reflective functioning (RF), and sensitivity in the parent-child interaction (Model 2).

| path | parent | ß | *b* [95% CI] | *se* | *p* |
| --- | --- | --- | --- | --- | --- |
| RF (T1), int. | F | 2.69** | 3.71 [3.25; 4.37] | 0.28 | <.001 |
| RF (T1)<--Secure AR (T1) | F | 0.7** | 2.05 [1.26; 2.75] | 0.37 | <.001 |
| RF (T1), int. | M | 3.03** | 3.98 [3.37; 4.55] | 0.31 | <.001 |
| RF (T1)<--Secure AR (T1) | M | 0.69** | 2.17 [1.49; 2.82] | 0.35 | <.001 |
| RF (T2), int. | F | 0.36 | 0.59 [-1.53; 2.63] | 1.06 | .576 |
| RF (T2)<--RF (T1) | F | 0.27 | 0.33 [-0.06; 0.83] | 0.23 | .147 |
| RF (T2)<--RF (T1) | F<-M | 0.22 | 0.28 [-0.14; 0.64] | 0.19 | .149 |
| RF (T2)<--Secure AR (T1) | F | 0.25 | 0.88 [-0.75; 2.34] | 0.77 | .252 |
| RF (T2), int. | M | 2.43** | 2.76 [1.14; 4.4] | 0.83 | .001 |
| RF (T2)<--RF (T1) | M | 0.34 | 0.29 [-0.26; 0.75] | 0.25 | .251 |
| RF (T2)<--RF (T1) | M<-F | 0.27 | 0.22 [-0.07; 0.57] | 0.16 | .166 |
| RF (T2)<--Secure AR (T1) | M | -0.08 | -0.22 [-1.09; 0.81] | 0.49 | .647 |
| Sensitivity (T2), int. | F | 3.68** | 4.22 [2.68; 5.94] | 0.84 | <.001 |
| Sensitivity (T2)<--RF (T2) | F | 0.78** | 0.54 [0.37; 0.72] | 0.09 | <.001 |
| Sensitivity (T2)<--RF (T2) | F<-M | -0.3 | -0.31 [-0.71; 0] | 0.18 | .085 |
| Sensitivity (T2), int. | M | 4.15** | 4.59 [2.76; 6.46] | 0.95 | <.001 |
| Sensitivity (T2)<--RF (T2) | M | 0.12 | 0.12 [-0.29; 0.48] | 0.19 | .546 |
| Sensitivity (T2)<--RF (T2) | M<-F | 0.15 | 0.1 [-0.14; 0.35] | 0.12 | .413 |
| Secure AR (T1), int. | F | 1.44** | 0.68 [0.5; 0.8] | 0.08 | <.001 |
| Secure AR (T1), int. | M | 1.86** | 0.78 [0.65; 0.9] | 0.07 | <.001 |
| RF (T2)<-->RF (T2) | F<->F | 0.63** | 1.73 [1.07; 2.96] | 0.44 | <.001 |
| RF (T2)<-->RF (T2) | M<->M | 0.77** | 1 [0.73; 1.38] | 0.19 | <.001 |
| RF (T1)<-->RF (T1) | F<->F | 0.51** | 0.98 [0.63; 1.55] | 0.21 | <.001 |
| RF (T1)<-->RF (T1) | M<->M | 0.53** | 0.91 [0.57; 1.41] | 0.20 | <.001 |
| Sensitivity (T2)<-->Sensitivity (T2) | F<->F | 0.54** | 0.71 [0.54; 1.06] | 0.13 | <.001 |
| Sensitivity (T2)<-->Sensitivity (T2) | M<->M | 0.95** | 1.16 [0.89; 1.59] | 0.18 | <.001 |
| Secure AR (T1)<-->Secure AR (T1) | F<->F | 1** | 0.22 [0.14; 0.25] | 0.03 | <.001 |
| Secure AR (T1)<-->Secure AR (T1) | M<->M | 1** | 0.17 [0.09; 0.23] | 0.04 | <.001 |
| RF (T2)<-->RF (T2) | F<->M | 0.34 | 0.45 [-0.01; 1.04] | 0.26 | .078 |
| RF (T1)<-->RF (T1) | F<->M | 0.47** | 0.44 [0.15; 0.79] | 0.16 | .006 |
| Secure AR (T1)<-->Secure AR (T1) | F<->M | 0.52** | 0.1 [0.03; 0.16] | 0.03 | .002 |
| Sensitivity (T2)<-->Sensitivity (T2) | F<->M | 0.23 | 0.21 [-0.04; 0.5] | 0.14 | .128 |
| h1a_father | h<-s | 0.12 | 0.31 [-0.11; 0.86] | 0.23 | .188 |
| h1a_mother | h<-s | 0.02 | 0.06 [-0.12; 0.44] | 0.13 | .665 |
| h1b_father | h<-s | 0.17 | 0.15 [-0.05; 0.37] | 0.11 | .171 |
| h1b_mother | h<-s | 0.03 | 0.03 [-0.05; 0.22] | 0.06 | .672 |
| h3_father | h<-r | 0.17 | 0.15 [-0.05; 0.37] | 0.11 | .171 |
| h3_mother | h<-r | 0.03 | 0.03 [-0.05; 0.22] | 0.06 | .672 |
| *ꭕ^2^(12)*=12.907 (p=0.376); *CFI*=0.993; *TLI*=0.984; *RMSEA*=0.043 95% CI [0; 0.17]; *N*=40 | | | | | |
| Note. F = father, M = mother. ß is the path coefficient with all variables standardized; *b* [95% CI] is the unstandardized path coefficient with a 95% bias-corrected and accelerated confidence interval; *se* is the bootstrapped standard error the estimate; int. is the intercept of the path; **p* < .05; ** *p* < .01; all calculations are based on 10.000 bootstrap replicates. *h1a_father* = Indirect effect of father attachment via father RF_AAI via father RF_PDI on father sensitivity; *h1a_mother* = Indirect effect of mother attachment via mother RF_AAI via mother RF_PDI on mother sensitivity; *h1b_father* = Indirect effect of father RF_AAI via father RF_PDI on father sensitivity; *h1b_mother* = Indirect effect of mother RF_AAI via mother RF_PDI on mother sensitivity; *h3_father* = Indirect effect of mother RF_AAI via father RF_PDI on father sensitivity; *h3_mother* = Indirect effect of father RF_AAI via mother RF_PDI on mother sensitivity. | | | | | |

**Table S4**. Results of the fully restrained structural equation model for attachment representation (AR), reflective functioning (RF), and sensitivity in the parent-child interaction (Model 3).

| path | parent | ß | *b* [95% CI] | *se* | *p* |
| --- | --- | --- | --- | --- | --- |
| RF (T1), int. | F | 2.71** | 3.78 [3.35; 4.24] | 0.22 | <.001 |
| RF (T1)<--Secure AR (T1) | F | 0.71** | 2.21 [1.69; 2.69] | 0.26 | <.001 |
| RF (T1), int. | M | 2.71** | 3.78 [3.35; 4.24] | 0.22 | <.001 |
| RF (T1)<--Secure AR (T1) | M | 0.71** | 2.21 [1.69; 2.69] | 0.26 | <.001 |
| RF (T2), int. | F | 0.96* | 1.45 [0.24; 2.98] | 0.70 | .038 |
| RF (T2)<--RF (T1) | F | 0.43** | 0.46 [0.18; 0.84] | 0.17 | .007 |
| RF (T2)<--RF (T1) | F<-M | 0.13 | 0.14 [-0.03; 0.32] | 0.09 | .133 |
| RF (T2)<--Secure AR (T1) | F | 0.08 | 0.26 [-0.92; 1.18] | 0.51 | .611 |
| RF (T2), int. | M | 0.96* | 1.45 [0.24; 2.98] | 0.70 | .038 |
| RF (T2)<--RF (T1) | M | 0.43** | 0.46 [0.18; 0.84] | 0.17 | .007 |
| RF (T2)<--RF (T1) | M<-F | 0.13 | 0.14 [-0.03; 0.32] | 0.09 | .133 |
| RF (T2)<--Secure AR (T1) | M | 0.08 | 0.26 [-0.92; 1.18] | 0.51 | .611 |
| Sensitivity (T2), int. | F | 3.13** | 3.71 [2.6; 4.7] | 0.54 | <.001 |
| Sensitivity (T2)<--RF (T2) | F | 0.55** | 0.43 [0.28; 0.58] | 0.07 | <.001 |
| Sensitivity (T2)<--RF (T2) | F<-M | -0.13 | -0.1 [-0.29; 0.05] | 0.09 | .239 |
| Sensitivity (T2), int. | M | 3.13** | 3.71 [2.6; 4.7] | 0.54 | <.001 |
| Sensitivity (T2)<--RF (T2) | M | 0.55** | 0.43 [0.28; 0.58] | 0.07 | <.001 |
| Sensitivity (T2)<--RF (T2) | M<-F | -0.13 | -0.1 [-0.29; 0.05] | 0.09 | .239 |
| Secure AR (T1), int. | F | 1.62** | 0.72 [0.57; 0.83] | 0.06 | <.001 |
| Secure AR (T1), int. | M | 1.62** | 0.72 [0.57; 0.83] | 0.06 | <.001 |
| RF (T2)<-->RF (T2) | F<->F | 0.69** | 1.58 [1.14; 2.26] | 0.29 | <.001 |
| RF (T2)<-->RF (T2) | M<->M | 0.69** | 1.58 [1.14; 2.26] | 0.29 | <.001 |
| RF (T1)<-->RF (T1) | F<->F | 0.5** | 0.96 [0.69; 1.27] | 0.15 | <.001 |
| RF (T1)<-->RF (T1) | M<->M | 0.5** | 0.96 [0.69; 1.27] | 0.15 | <.001 |
| Sensitivity (T2)<-->Sensitivity (T2) | F<->F | 0.73** | 1.03 [0.81; 1.39] | 0.13 | <.001 |
| Sensitivity (T2)<-->Sensitivity (T2) | M<->M | 0.73** | 1.03 [0.81; 1.39] | 0.13 | <.001 |
| Secure AR (T1)<-->Secure AR (T1) | F<->F | 1** | 0.2 [0.14; 0.24] | 0.03 | <.001 |
| Secure AR (T1)<-->Secure AR (T1) | M<->M | 1** | 0.2 [0.14; 0.24] | 0.03 | <.001 |
| RF (T2)<-->RF (T2) | F<->M | 0.13 | 0.2 [-0.3; 0.84] | 0.28 | .466 |
| RF (T1)<-->RF (T1) | F<->M | 0.4** | 0.39 [0.11; 0.73] | 0.15 | .008 |
| Secure AR (T1)<-->Secure AR (T1) | F<->M | 0.5** | 0.1 [0.02; 0.16] | 0.03 | .003 |
| Sensitivity (T2)<-->Sensitivity (T2) | F<->M | 0.26 | 0.27 [-0.02; 0.64] | 0.16 | .101 |
| h1a | h<-s | 0.17* | 0.44 [0.17; 0.91] | 0.18 | .014 |
| h1b | h<-s | 0.23** | 0.2 [0.08; 0.4] | 0.08 | .009 |
| h3 | h<-r | 0.07 | 0.06 [-0.01; 0.14] | 0.04 | .125 |
| *ꭕ^2^(26)*=48.964 (p=0.004); *CFI*=0.824; *TLI*=0.811; *RMSEA*=0.149 95% CI [0.082-0.212]; *N*=40 | | | | | |
| Note. F = father, M = mother. ß is the path coefficient with all variables standardized; *b* [95% CI] is the unstandardized path coefficient with a 95% bias-corrected and accelerated confidence interval; *se* is the bootstrapped standard error the estimate; int. is the intercept of the path; **p* < .05; ** *p* < .01; all calculations are based on 10.000 bootstrap replicates. *h1a* = Indirect effect of attachment via RF_AAI via RF_PDI on sensitivity; *h1b* = Indirect effect of RF_AAI via RF_PDI on sensitivity; *h3* = Indirect effect of parents RF_AAI via coparents RF_PDI on coparents sensitivity. | | | | | |

**Figure S1***.* Proposed Actor-Partner Interdependence Model


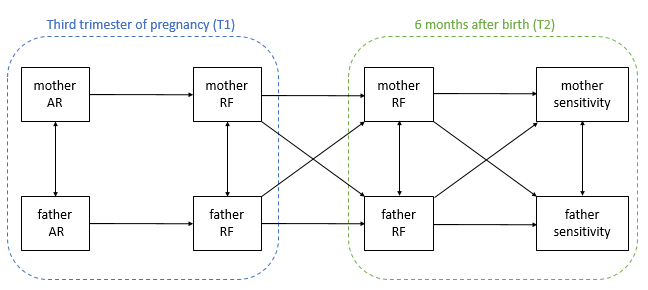


Note. AR = adult attachment representations; RF = reflective functioning

**Figure S2***.* Actor and partner effects for reflective functioning (RF) at T1 and T2.


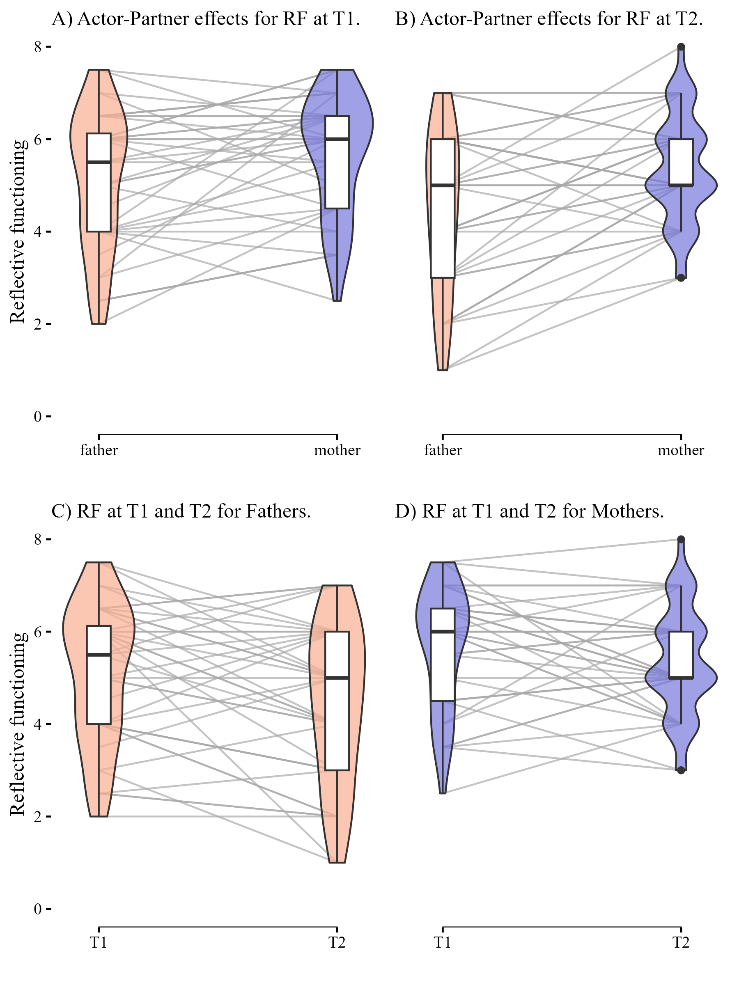


**Figure S3**. Boxplots and bar-charts for visual comparison of dropouts.


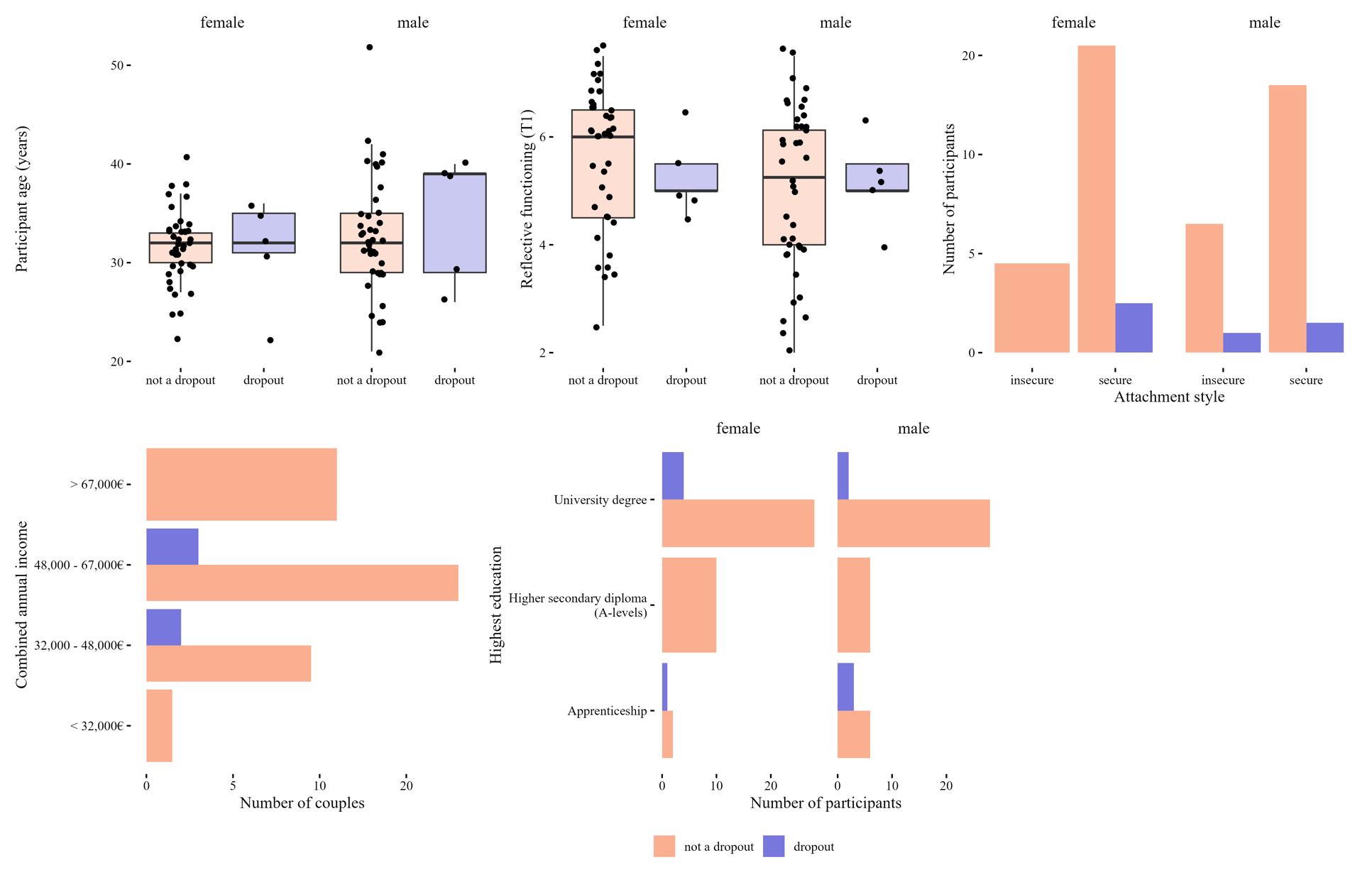

Supplement: Supplementary file 1 [file Supplementary_file_1.docx]
